# Stroke in Middle Eastern children with cancer: prevalence and risk factors

**DOI:** 10.1186/s12883-022-02556-x

**Published:** 2022-01-18

**Authors:** Catherina Zadeh, Natally AlArab, Samar Muwakkit, Lamya A. Atweh, Hani Tamim, Maha Makki, Hamza A. Salhab, Roula Hourani

**Affiliations:** 1 Department of Diagnostic Radiology, American University of Beirut Medical Center, Beirut, Lebanon; 2 Department of Pediatrics and Adolescent Medicine, Children’s Cancer Center of Lebanon, American University of Beirut Medical Center, Beirut, Lebanon; 3 Biostatistics Unit, Clinical Research Institute, American University of Beirut Medical Center, Beirut, Lebanon

## Abstract

**Objective:**

To determine the prevalence and to characterize the different types of strokes in children with cancer at the Children’s Cancer Center of Lebanon (CCCL), in addition to assess the factors and clinical findings leading to stroke in children.

**Methods:**

We retrospectively reviewed the medical records and brain images (MRIs and CTs) of children admitted to the CCCL and diagnosed with cancer between years 2008 and 2017. Brain images were reviewed for the strokes’ onset, size, location, possible origin, its recurrence and type: intracranial hemorrhage (ICH), acute arterial ischemic stroke, and cerebral sinus venous thrombosis (CSVT) with and without venous infarct. Medical charts of the patients were reviewed for age, sex, their type of cancer, the treatment protocol they followed, and abnormal findings on their laboratory studies and neurological exams.

**Results:**

Out of the 905 charts reviewed, twenty-seven children with variable types of cancer had strokes, with a prevalence of 2.9%. Their median age at cancer diagnosis was 9.4 (4.8-13.7) years and the median age at stroke onset was 10.6 (6.7-15.5) years. The median time between the cancer diagnosis and the stroke episode was 6 months. CSVT cases were the most common (60%) followed by acute arterial ischemic (22%) and hemorrhagic strokes (18%), with CSVT being the latest to occur. We observed that the different types of strokes were related to some types of cancer. Of the children that had acute arterial ischemic stroke in this cohort, 83% had brain tumors, of the children who had CSVT, 87.5% had leukemia, and of the children who had hemorrhagic stroke, 40% had leukemia. Neurological abnormalities were more prevalent in acute arterial ischemic stroke (80%). Patients with CSVT recovered better than those with other types of strokes. Strokes recurred in 60% of ischemic strokes. L-Asparaginase was significantly associated with CSVT.

**Conclusions:**

The prevalence of strokes was 2.9% in children with cancer. We were able to identify factors related to the types of the stroke that occurred in children including the type and location of the cancer the type of treatment received, and stroke recurrence.

## Introduction

Stroke is a serious complication of malignancy in children leading to increased morbidity and higher need for critical services [1]. Stroke has a prevalence of approximately 1% in children with cancer, with equal incidence of both acute arterial ischemic and hemorrhagic strokes. Among those, children with leukemia and brain tumor were found to have the highest risk for stroke [2]. Recent studies reveal that adult survivors of childhood cancer had an increased risk of cerebrovascular disease [3]. However, little is known about strokes within the first 5 years from the onset of cancer during childhood [2]. There is limited data on the epidemiology of strokes in children including the incidence, types, characteristics, and factors contributing to the strokes in child cancer survivor. It is important for clinicians managing pediatric cancer patients to be aware of the likelihood of a stroke happening for the type of cancer they are treating and risk factors leading to it. This will allow the early detection and treatment of stroke cases.

Many factors have been associated with strokes in children with cancer. The pathophysiology of the stroke is either mediated directly by the cancer itself or indirectly by an induced hypercoagulable state [1]. The literature suggests that the type of cancer in addition to whether the patient received cranial radiation therapy are strong predictors of the incidence of first stroke in cancer patients [4]. The use of chemotherapy, especially L- asparaginase, is a known risk factor for venous thromboembolism including cerebral sinus venous thrombosis (CSVT) leading in one-third of the cases to parenchymal hemorrhage [5] and neurological deficits [6]. Bowers et al. showed that thirty-seven leukemia survivors and sixty-three brain tumor survivors reported a late-occurring stroke with a relative risk of 6.4 (95% CI, 3.0 to 13.8; *P*<0.0001) and 29.0 (95% CI, 13.8 to 60.6; *P*<0.0001) for leukemia and brain tumor survivors respectively [4].

The purpose of this study is to retrospectively review the demographic characteristics of children with cancer treated at the Children Cancer Center of Lebanon (CCCL). This will allow us to determine the prevalence of stroke in addition to characterizing the types of strokes and assessing the factors and clinical findings that might have contributed to the stroke by comparing the clinical and radiological characteristics between children with different types of stroke.

## Materials and methods

### Subjects

The study included all children admitted to the Children Cancer Center of Lebanon (CCCL) between January 2008 and December 2017 aged 18 years and younger with a total of 905 cases. These patients were diagnosed with different types of cancer including solid tumors, leukemia, or lymphoma; and have undergone brain imaging (MRI and CT scan) showing positive stroke findings in their reports.

### Data collection

We retrospectively extracted all the brain images (including MRIs and CTs) of the 905 children from the PACS imaging system and reviewed their corresponding reports for the presence of stroke: intracranial hemorrhage (ICH), acute arterial ischemic stroke, and cerebral sinus venous thrombosis (CSVT) with and without venous infarct. Children with intratumoral hemorrhage, traumatic hemorrhage, subdural and epidural hemorrhage, and bleeding related to surgical interventions were excluded. We also collected data on the patients’ clinical presentation, history and laboratory test results. All previous and follow up imaging studies were also reviewed until the child’s last admission.

Data collected from the patients’ charts included: gender, age at cancer diagnosis, age at stroke diagnosis, time from cancer diagnosis to stroke onset, stroke type (ICH, acute arterial ischemic stroke, CSVT with and without venous infarct), cancer type (brain tumor, lung tumor, leukemia, lymphoma), and radiological reports finding such as size of stroke (small versus large). Acute arterial ischemic stroke was defined as acute neurologic deficits lasting more than 24 h and caused by cerebral ischemia secondary to sudden loss of blood circulation in a vascular territory; associated with neuroimaging showing parenchymal infarction conforming to known arterial territories and corresponding to the clinical presentation. Regarding the middle cerebral artery (MCA) and posterior circulation arterial ischemic strokes, we referred to the ASPECT Score and pc ASPECT scoring: less than 1/3 of the territory (score 7) was considered small and below 7 is large [7]. A hemorrhagic stroke occurs when a blood vessel in the brain or on the surface of the brain leaks causing bleeding which can lead to brain tissue damage with secondary acute neurologic deficit lasting more than 24 h [8]. We considered the hemorrhagic stroke large if its size was above > 50-60 ml CSVT referred to patients who had thrombosis with or without venous infarcts including secondary hemorrhagic transformations. All lacunar infarcts measuring less than 1.5 cm were classified as small. We defined the location of stroke including the basal ganglia, lobar or posterior fossa. The lobar strokes were located superficially within the lobes of the brain. We tried to identify the possible origin of the stroke (venous or arterial), when possible. We also reviewed the treatment regimens used including surgical treatment, radiation, type of chemotherapy, if present, and any other drug. The risk factors for stroke were evaluated, such as abnormal platelet counts and leukocyte counts. Abnormal coagulation studies at the time of the stroke were noted for any abnormal values including: prothrombin time (PT) (normal range 10-30 s), partial thrombin time (PTT) (normal range 30-45), and international normalized ratio (INR) normal (range between 1 and 2). The recorded neurological status at diagnosis and at the last follow up were reviewed. Finally, the recurrence of the stroke as well as its outcome were recorded.

The denominator for period prevalence (how many children with cancer had stroke during the study period) was the number of pediatric patients that were admitted to the CCCL during the study period. An experienced neuroradiologist with expertise in stroke reviewed the extracted brain images to confirm the strokes, and a pediatric oncologist reviewed all the extracted clinical information.

### Statistical analysis

Analysis was performed using SPSS version 24. Continuous data was reported as medians and 25%tile and 75%tile QR, and were compared between acute arterial ischemic stroke, hemorrhagic stroke and CSVT groups using the independent sample median test. Categorical data was reported as numbers and percentages and was compared using the Chi-Square test. A p-value < 0.05 was considered statistically significant.

## Results

### Demographics

By searching the database of CCCL patients, we were able to identify 905 children with cancer during our 10-year study period. A total of 27 patients diagnosed with stroke were included in the study. The cancer types of these patients included seven brain tumor cases (glioblastoma multiforme, craniopharyngioma, grade I astrocytoma, 2 cases of grade II pilomyxoid astrocytoma and 2 cases of medulloblastoma), one lung cancer (pulmonary blastoma), 17 leukemia cases (6 T-cell acute lymphoblastic leukemia (ALL), 6 Pre-B cell ALL, 3 B-cell ALL, 1 acute prolymphocytic leukemia, and 1 non specified type) and 2 B-cell lymphoma cases. There were 19 males and 8 females with a median age of 9.4 (4.8-13.7) years at cancer diagnosis and 10.6 (6.7-15.5) years at the time of stroke diagnosis (Table 1). There were six patients with acute arterial ischemic stroke (22%), five patients with hemorrhagic stroke (18%), and 16 patients with CSVT with no infarcts (60%). Three of the 16 patients with CSVT developed hemorrhagic venous stroke later on.

**Table 1:** Demographic and clinical characteristics of children with cancer presenting in acute ischemic, hemorrhagic and cerebral sinus venous thrombosis (CSVT)

Characteristics	Total *N*=27	Stroke type			*P* value
		**CSVT** ***N*=16**	**Hemorrhagic** ***N*=5**	**Acute Ischemic** ***N*=6**	
Age at cancer diagnosis (years)	9.4 (4.8-13.7)	9.4 (5.5-14.7)	10.6 (4.9-12.8)	5.7 (3.6-12.7)	0.68
Age at stroke diagnosis (years)	10.6 (6.7-15.5)	11.4 (6.1-15.7)	10.6 (6.5-12.8)	10.3 (6.2-16.4)	0.92
Stroke onset after Cancer diagnosis: (months)	6 (1-9)	8 (5-9)	1 (0.1-19)	1.8 (0.2-39)	0.58
Time to follow-up (years)	5 (4-6)	5 (4.3-6)	4 (2-6)	4.5 (2.5-7)	0.77
Sex (Male)	19 (70.4%)	11 (68.8%)	2 (40%)	1 (16.7%)	0.73
Cancer type					0.001
Brain	7 (25.9%)	1 (6.3%)	1 (20%)	5 (83.3%)	
Leukemia	17 (63.0%)	14 (87.5%)	2 (40%)	1 (16.7%)	
Lymphoma	2 (7.4%)	1 (6.3%)	1 (20%)	0	
Lung	1 (3.7%)	0	1 (20%)	0	
Stroke size					0.56
Small	8 (72%)	-	4 (80%)	4 (66.7%)	
Large	3 (28%)	-	1 (20%)	2 (33.3%)	
Location of stroke					<0.0001
Basal ganglia	3 (17.6%)	-	1 (20%)	2 (33.3%)	
Lobar^a^	4 (14.8%)	-	2 (40%)	2 (33.3%)	
Posterior fossa	4 (23.5%)	0	2 (40%)	2 (33.3%)	
Treatment of cancer Drugs Surgical Combined	19 (70.4%) 1 (3.7%) 7 (25.9%)	15 (93.8%) 0 1 (6.3%)	3 (60%) 0 2 (40%)	1 (16.7%) 1 (16.7%) 4 (66.7%)	0.002
Leukocyte count (×10^3^ /mm^3^)	6.3 (3.8-35.3)	4.5 (3.6-51.3)	15.3 (6-134)	7.8 (4.5-24.2)	0.30
Platelet count (×10^3^ /mm^3^)	205 (141-318)	179 (143.8-287.3)	170 (61.5-395)	303.5 (207-401)	0.147
Abnormal coagulation (yes)	14 (51.9%)	8 (50%)	3 (60%)	3 (50%)	1.00
Stroke recurrence (yes)	4 (19.0%)	0	1 (25.0%)	3 (60.0%)	0.01
Death (yes)	2 (7.7%)	0	1 (20%)	1 (17%)	0.14

^a^Lobar: located superficially within the lobes of the brain involving the cortex but not the basal ganglia

### Clinical features of strokes

In our study, the prevalence of stroke in children with new diagnosis of cancer over a 10 -year period was estimated to be 2.9%. Patients ranged in age between 2.5 months and 18 years, with a median age of 5.7 (3.6-12.7) years for acute ischemic stroke, 10.6 (4.9-12.8) years for hemorrhagic stroke, and 9.4 (5.5-14.7) years for CSVT. The median interval time between the dates of cancer diagnosis to the onset of stroke was 6 (1-9) months. Hemorrhaging strokes occur the earliest after cancer diagnosis, followed by acute ischemic strokes then CSVT (Table 1). At presentation, all patients had neurological symptoms including headache, visual impairment, dysarthria, and upper and/or lower motor spasms or weakness. Nine patients did not have any acute focal neurological deficits. The reason why these patients underwent imaging is the presence of different symptoms suggesting the presence of a neurological problem. The symptoms included: headaches (three patients), vomiting (three patients), dizziness (one patient), slurred speech (one patient) and decreased level of consciousness and petechia. Abnormal neurological exams were most frequent in acute arterial ischemic stroke patients (80%). The type of cancer diagnosed in patients seemed to affect the type of strokes they experienced (*p value = 0.001*). Of the children who had ischemic stroke in this cohort, 83% had brain tumors. Out of seven patients with brain tumor, only one had right MCA severe stenosis related to radiation vasculopathy, and six patients developed stroke as a post-operative complication. On the other hand, hemorrhagic stroke and CSVT were observed more frequently in leukemia patients (Table 1). There was a single case of lung cancer that later led to a hemorrhagic stroke. The stroke size was small in 62% of the cases and did not showing any statistical significance when comparing hemorrhagic and arterial ischemic strokes. Ischemic strokes were mainly arterial in origin whereas hemorrhagic strokes can be arterial in origin or secondary to venous thrombosis. There was no statistical difference in the location of strokes in basal ganglia, lobar or posterior fossa in hemorrhagic and arterial ischemic strokes. Among our cohort, abnormal coagulation parameters were present in 51% of the cases, where abnormal PT, PTT and INR values were evident, with no significant variation among the three stroke groups. The Leukocyte and platelet counts were not associated with any specific stroke category. Only 17 children were followed-up with imaging (15 CSVT cases and 2 ischemic strokes) as was clinically indicated. Three patients were lost to follow up. The median time of last follow up in relationship to stroke presentation was 5 (4-6) years. Strokes were recurrent in 60% of acute arterial ischemic strokes, whereas only 25% of hemorrhagic strokes re-occurred. One patient with ischemic stroke and another one with hemorrhagic stroke died 8 to 19 months after stroke onset. The neurological status at the last follow up visit was analyzed and it showed improvement in most CSVT cases (86.7%) and some of the hemorrhagic stroke children (75%). However, all the children with ischemic strokes had a persistent abnormal neurological status at their latest follow up visit.

### Treatment and stroke subtype

In our cohort, the stroke type differed based on the type of treatment. Cancer patients treated with chemotherapy were most likely to develop CSVT and hemorrhagic stroke rather than arterial ischemic strokes. Patients treated with combined therapy (surgical intervention, radiotherapy and chemotherapy) were more likely to develop arterial ischemic strokes (p-value=0.002). We compared the frequencies of each stroke type in the different types of chemotherapies used in order to determine which chemotherapeutic drug was most likely to be associated with stroke incidence. Drugs recorded were mainly chemotherapies and suppressors such as vincristine, L-asparginase, cyclophosphamide, doxorubicin, cisplatin, etoposide, etc. Our results showed that 100% of the patients who developed CSVT were taking L-asparginase in their treatment, and none of these patients has developed primary hemorrhagic or ischemic strokes not secondary to the CSVT (p *value <0.0001*). The frequency of ischemic arterial strokes were higher (three out of five) in vincristine-treated cases compared to other drugs (two out of twenty).

## Discussion

In our study, the prevalence of stroke in 905 children diagnosed with cancer over a 10-year period was estimated to be 2.9% with a median time interval from the date of cancer diagnosis to date of stroke episode being 6 months. This prevalence was higher than those seen in the literature, but with similar time of onset. A study performed by Noje et al. showed a prevalence of 1.1% with strokes occurring at a median of 5 months after cancer diagnosis. This difference could be related in part to the higher population sample (more than 1,400 children compared to 905 in our study) [2]. Another study performed by Parasole et al. showed a prevalence of strokes estimated at 1.97% in children treated for ALL [9]. In this study, CSVT were not included, which might explain the lower prevalence of strokes in their study when compared to ours. Similarly, Santoro et al. reported a prevalence of 0.47% from a total of 2,318 ALL cases; however all the strokes were found to be CSVT [10]. DiMario et al. retrospectively reviewed 815 children with systemic cancer (excluding primary central nervous system tumors) over a 6-year period and showed a prevalence of 1.5% of strokes [11]. This lower prevalence might be explained by the exclusion of brain tumors from the study. On the other hand, Packer et al. reported 4% of patients (26 out of 700 cases) having cerebrovascular accidents over a period of 4 years. It is worth mentioning that the diagnosis of cerebrovascular accidents used by Packer et al. was not only based on brain scan findings but also on postmortem histopathological examination which was performed in 54 of 295 (18%) children with cancer who died during the time period of the study [12]. The prevalence comparison represents an estimate of the true numbers since the values differ based on the detection rates as well as the type of strokes included in each study as detailed above. Male predominance was evident in our cohort. This finding was concordant with Noje et al. which showed that 60% of the stroke positive patients were males [2].

CSVT appeared to be the most frequent type of strokes in our study, a result discordant with the finding of Noje et al. who showed that intracranial hemorrhagic (ICH) strokes are more common [2]. This can be explained by the difference in the predominance of cancer types. In our study, 17 out of 27 patients had leukemia, compared to 8 patients out of 15 in the study by Noje et al. Patients with leukemia are at higher risk for developing CSVT due to the type of chemotherapy treatment. In addition to that, the type of cancer showed significant difference between each group of strokes. Of the children who had ischemic stroke in this cohort, 83% had brain tumors, while hemorrhagic strokes and CSVT were more frequently seen in children with leukemia. Our results were concordant with the literature, where 75% of stroke patients with brain tumors were ischemic in nature compared to 71.4% and 66.7% for ICH and CSVT respectively in patients with leukemia [2].

Cancer treatments, such as radiation therapy and L-ASPA among many others, are known risk factors for cerebral strokes. In patients with brain tumors, the major risk factors for arterial ischemic stroke are treatment or disease related, such as tumor invasion of the vessel, direct vascular injury related to the procedure, and vasospasm secondary to sub-arachnoid hemorrhage [13]. Those factors explain the early reported onset of strokes in these patients directly after their diagnosis or in the post-operative period. Radiation induced arteriopathy, defined as abnormal findings on MRA, is a leading cause of stroke in pediatric patients with brain tumor. Another study proposed a correlation between higher radiation doses and an earlier time of onset of intracranial arteriopathy in the pediatric brain tumor population. The cumulative incidence of arteriopathy following cranial radiation therapy at 5 years and 10 years was 5.4% (95% CI 0.6%-10%) and 16% (95% CI 4.6%-26%), respectively [14].

Many factors were reported as possible causes for cerebral venous thrombosis in pediatric leukemia patients including coagulopathy secondary to disseminated intravascular coagulation, infection adjacent to the sinus, compression or infiltration by the tumor, and inherited deficiency of proteins C and S [15, 16]. L-asparaginase treatment has been found to be a cause of cerebral venous thrombosis due to a decline in the levels of anticoagulant proteins including protein C and S as well as anti-thrombin, which impairs thrombin inhibition, causing secondary platelet and endothelial activation. [17]. A study showed that the most important factor in ALL patients who develop cerebral sinus venous thrombosis is the intensive therapy and prolonged exposure to L-asparginase especially in patients with inherited thrombophilia [18].

Concerning the outcomes of our patients, improvement was seen in most CSVT cases (86.7%) and some of the hemorrhagic stroke cases (66.7%). Similar results were obtained in Santoro et al. where 91% of his included cases who developed CSVT had favorable neurological outcomes [10]. On the other hand, all the children with ischemic strokes had persistent abnormal neurological status at their latest follow up visit. When it comes to death rate, none of the CSVT patients died unlike the other two categories. Although two patients died after a hemorrhagic stroke and ischemic strokes respectively, yet the strokes cannot be considered a direct cause behind their death especially that the death incidence was not directly following the stroke diagnosis in these patients. Our results were not concordant with the results from the single-center study of 15 patients by Noje et al. in which the death rate was 75% in patients with ischemic strokes, 100% in patients with hemorrhagic strokes, and 66.7% in patients with CSVT [2]. These findings highlight the importance of early diagnosis and treatment of CSVT because it is reversible and is usually associated with less severe neurological outcomes.

One strength of this study is that all brain images were reviewed by a senior radiology resident by an experienced neuroradiologist to confirm that all strokes mentioned in the reports were positive and that the stroke location corresponds to the reported clinical manifestation in the patient chart. Moreover, we highlighted the type and location of strokes encountered in our pediatric population, which was not mentioned in previously conducted studies. This study was limited by its retrospective nature. The use of ICD-10 code searches may be inaccurate since the definitive diagnosis that can be coded using ICD is achieved after several patient visits and rarely during the first visit [19]. In addition, the small sample size of our cohort and the small samples in each subtype of stroke are a limitation. This can be due to the rarity of pediatric stroke in children with cancer and to the setting of the study, which was based on cases from a single cancer center. However, multicenter studies have been limited by patient-reported strokes and by the lack of imaging and medical record review.

To our knowledge, this is the first study conducted in Lebanon and the MENA region that looks at the prevalence of stroke and the risk factors associated with it among children with cancer. Knowing that CCCL is a leading cancer center in the MENA region, where a variety of cases are admitted from different ethnicities and backgrounds, we believe in the strength of this study. We believe that this study highlights the importance of assessing the risk factors that cancer patients might have and adjusting the treatment accordingly to lower the risk of stroke in future practice.

## Conclusions

Our conclusion was based on the results obtained in a small cohort of pediatric cancer patients who experienced strokes. We conclude that, although rare with a prevalence of 2.9%, strokes are one of the complications that can affect children with cancer. Ischemic strokes affect patients with brain tumors more frequently compared to other cancer types, whereas CSVT and hemorrhagic strokes are more common in leukemia patients compared to the other cancer types included in the study. Patients on chemotherapy were most likely to develop CSVT and hemorrhagic stroke rather than ischemic strokes. In addition, we observed that L-asparaginase was associated with cerebral sinus venous thrombosis. The obtained demographic and clinical results will allow the treating physician to diagnose stroke in children with cancer, taking into consideration the risk factors and the neurological deficits. In turn this will allow early diagnosis, prompt management and thus better clinical outcome.

## Data Availability

The data that support the findings of this study are available from the American University of Beirut Medical Center. Restrictions apply to the availability of these data, which were used under license for this study. Data are available from the corresponding author with the permission of our institution.
